# Mechanical strength of stainless steel and titanium alloy mini-implants with different diameters: an experimental laboratory study

**DOI:** 10.1186/s40510-021-00352-w

**Published:** 2021-03-22

**Authors:** Sérgio Estelita Barros, Viviane Vanz, Kelly Chiqueto, Guilherme Janson, Eduardo Ferreira

**Affiliations:** 1 Division of Orthodontics, Faculty of Dentistry, Federal University of Rio Grande do Sul, Rua Ramiro Barcelos 2492, Porto Alegre, RS 90035-003 Brazil; 2 Department of Orthodontics, Bauru Dental School, University of São Paulo, Alameda Octávio Pinheiro Brisolla 9-75, Bauru, SP 17012-901 Brazil

**Keywords:** Mini-implant, Orthodontic anchorage, Flexural strength, Torsional strength, Stainless Steel, Titanium alloy

## Abstract

**Background:**

The mechanical strength of mini-implants is a critical factor due to their small diameters. Currently, it is not possible to state whether there is a relevant difference between the mechanical properties of stainless steel (SS-MIs) and titanium alloy mini-implants (TA-MIs). The objective of this study was to test the null hypothesis that there is no difference in the mechanical strength of SS-MIs and TA-MIs, and to analyze, by scanning electron microscopy (SEM), the SS-MI, and TA-MI threads resistance to morphological damage after insertion.

**Methods:**

A standardized sample of 504 SS-MIs and TA-MIs with diameters ranging from 1.2 mm to 1.8 mm was used. Torsional fracture was performed in 154 MIs. Flexural strength of 280 MIs was evaluated at 1 mm and 2 mm-deflection. The threads of 70 MIs were morphologically analyzed by scanning electron microscopy (SEM), before and after their insertion in high-density artificial bone blocks. Comparisons between SS-MIs and TA-MIs were performed with *t* tests or Mann-Whitney *U* tests. A multiple linear regression analysis was used to evaluate the influence of variables on the ranging of MI mechanical strength.

**Results:**

SS-MIs had higher fracture torque. The mean difference between the SS-MIs and TA-MIs fracture torque was of 4.09 Ncm. The MI diameter explained 90.3% of the total variation in fracture torque, while only 2.2% was explained by the metallic alloy. The SS-MI group presented a higher deformation force during the 1mm and 2mm-deflection. The mean difference between the flexural strength of SS and TA-MIs at 1 mm and 2 mm-deflection was of 18.21 N and 17.55 N, respectively. There was no noticeable morphological damage to the threads of SS-MIs and TA-MIs.

**Conclusions:**

The null hypothesis was rejected. SS-MIs were 13.2% and 20.2% more resistant to torsional fracture and deflection, respectively. The threads of the SS-MIs and TA-MIs were not damaged during the insertion and removal process. Thus, the use of SS-MI can reduce the fracture risk without increasing the MI diameter.

## Background

Mini-implants (MIs) are advantageous supports for orthodontic treatment. Although MI use involves low risk, accidents and complications can occur. MIs with smaller diameters can be used to reduce the risk of injury to adjacent anatomical structures, especially in narrow interradicular septa. However, the decrease in diameter reduces MI structural strength, making it more susceptible to fracture and deflection [1, 2]. Mini-implant fracture may be difficult to clinically manage, making it a more severe complication than root contact [3–7].

Titanium alloy mini-implants (TA-MIs; Ti-6Al-4V) are the most widely used because it has higher mechanical strength than commercially pure titanium and is best suited to the small diameter of MIs, reducing the fracture risk during insertion and removal [8–10]. Lately, the use of stainless steel mini-implants (SS-MIs) for orthodontic anchorage have become more widespread among orthodontists and mini-implant manufacturers. However, there are few studies comparing mechanical properties and clinical performance of SS-MIs in relation to TA-MIs. A study evaluated the mechanical characteristics of SS-MIs comparing the results with the TA-MIs data from the literature [11]. Only two studies compared SS-MIs and TA-MIs, but they evaluated different MI brands and designs, compromising MI sample standardization [12, 13]. The results of these studies did not point in the same direction, perhaps due to a large variation in the MI samples [2, 14]. One of these studies showed that SS-MIs and TA-MIs had similar mechanical strength [12], while the other two studies found that SS-MIs were more resistant to mechanical failure than TA-MIs [11, 13].

Considering that SS-MIs and TA-MIs seems to have similar clinical efficiency [15–22], the use of more resistant MIs could be clinically interesting when insertion sites require a higher mechanical strength due to bone quality or availability (e.g., thick mandibular buccal shelf or narrow interradicular site limiting MI diameter). However, it is currently not possible to state whether there is a statistically and clinically relevant difference between the mechanical properties of SS-MIs and TA-MIs. Despite this, some articles have advocated the use of stainless steel mini-implants to obtain greater mechanical strength and prevent fractures [2, 16, 17, 19, 23]. In order to shed some light on this issue, the objective of this study was to test the null hypothesis that there is no difference in the mechanical strength of SS-MIs and TA-MIs.

## Methods

**Fig. 1:**
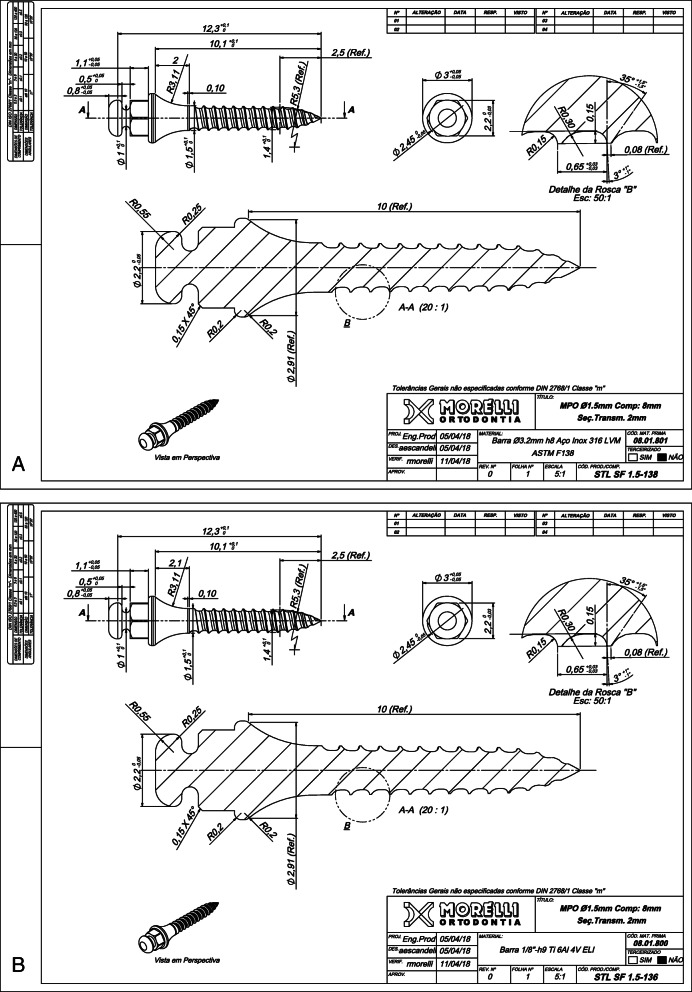
Morphological characteristic of mini-implants. Design for production used to obtain the standardization of morphologic and dimensional characteristics of the SS-MIs (**a**) and TA-MIs (**b**)

This was an experimental laboratory study conducted at the School of Dentistry, Federal University of Rio Grande do Sul. A standardized MI sample was specially manufactured for this study, so that the diameter and metallic alloy were the only differences between the MIs. A total of 504 self-drilling, tapered, 8-mm-long MIs with diameters ranging in increments of 0.1 mm from 1.2 to 1.8 mm (Fig. 1) was made of SS (252 MIs) and TA (252 MIs) (Dental Morelli, Sorocaba, SP, Brazil). Sample size calculation was performed assuming the values of 5% and 20% for α (type I error) and β (type II error), respectively. The minimum difference to be detected in the evaluation of torsional and flexural strength was 1 N/cm and 2 N, respectively. The assumed value of the variance of these measurements σ^2^ (standard deviation) was based on previous studies [1, 24]. Considering these parameters, torsional and flexural tests required a sample with a minimum of 11 and 10 MIs in each group, respectively. In addition, five SS-MI and five TA-MI of each diameter were added for qualitative morphological analysis of the MI threads by scanning electron microscopy, before insertion and after removal, in artificial bone blocks.

**Fig. 2:**
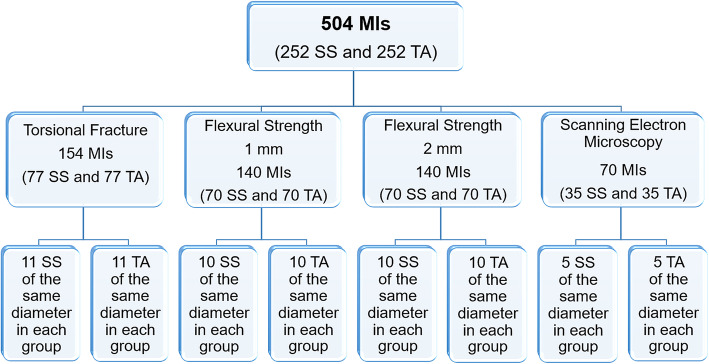
Flowchart of MI sample distribution. Five hundred and four self-drilling, tapered, 8-mm-long MIs with diameters ranging in increments of 0.1 mm from 1.2 to 1.8 mm (Fig. 1) was made of SS (252 MIs) and TA (252 MIs)

Distribution of the 504 MIs is shown in the flowchart (Fig. 2). Torsional fracture testing was performed on 154 MIs equally distributed into 14 groups (11 MIs per group) and allocated according to material (SS and TA) and diameter (1.2 mm to 1.8 mm) criteria. A total of 280 MIs was used to evaluate the flexural strength of SS-MIs and TA-MIs. These MIs were equally distributed into 28 groups (10 MIs per group) and allocated according to material (SS and TA), diameter (1.2 mm to 1.8 mm) and amount of deformation (1 mm and 2 mm) criteria. For microscopic analysis, a total of 70 MIs was equally distributed into 14 groups (5 MIs per group) and allocated according to material (SS and TA) and diameter (1.2 mm to 1.8 mm) criteria. Torsional, flexural, and microscopic evaluation of the MIs were performed as described ahead.

**Fig. 3:**
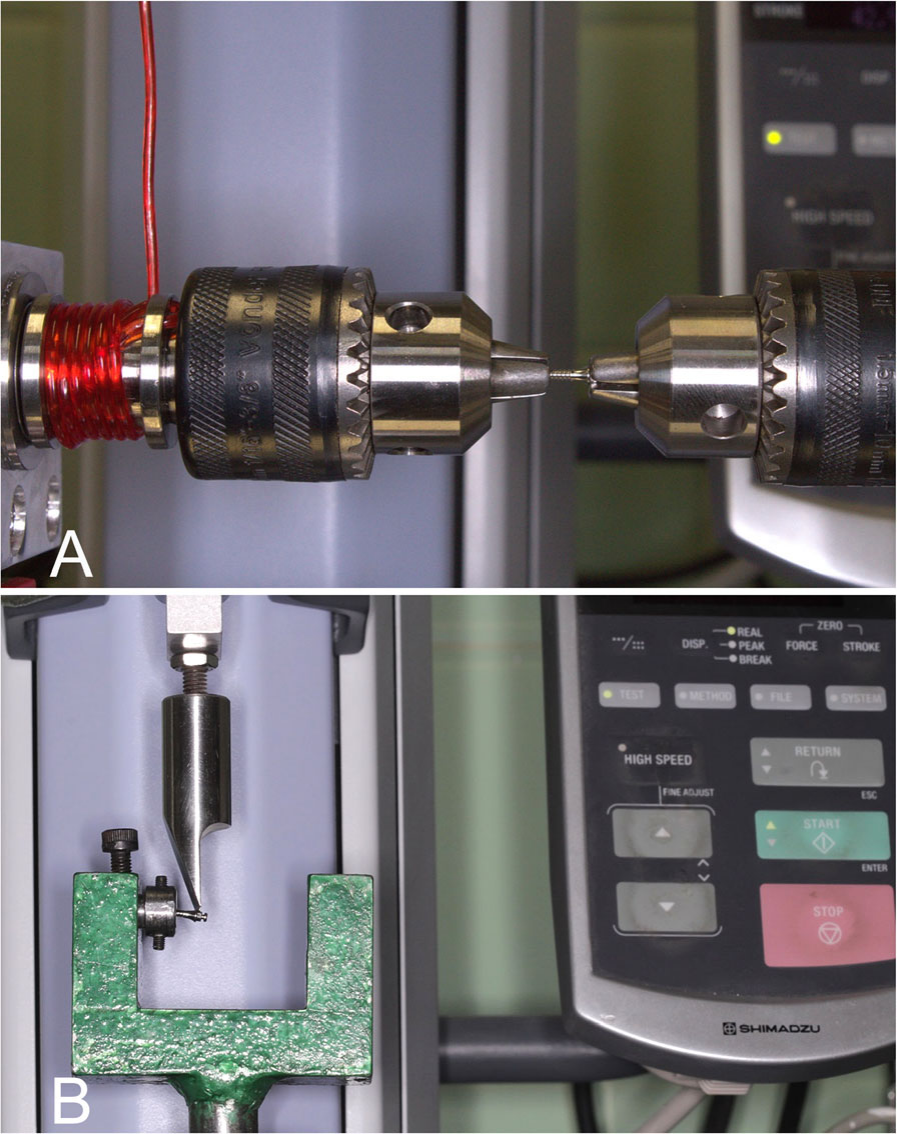
Custom-fabricated devices. Torsional (**a**) and flexural (**b**) tests using a mechanical testing machine (EZ-SX/Shimadzu, Barueri, SP, Brazil) with a 500 N load cell

Each of the 154 MIs specimen was coupled to the mechanical testing machine (EZ-SX/Shimadzu, Barueri, SP, Brazil) with a 500 N load cell, through a custom-fabricated device (Fig. 3a) for torsional loading test (Odeme Dental Research, Luzerna, SC, Brazil).The MI head was attached to a fixed jaw, horizontally aligned with a movable jaw, supported by ball bearings for rotation (Fig. 3a). The MI threads were gripped in the movable jaw 3 mm before the transmucosal collar (Fig. 3a). The movable jaw was turned by pulling a polymer wire wrapped around its axis and attached to the crosshead of the mechanical testing machine. The pull speed was set at 10 mm/min to produce a rotation speed of 90° per minute, increasing the torsional moment (units of N.cm) until MI fracture [8].

A custom-fabricated device (SENAI-CETEMP, São Leopoldo, RS, Brazil) coupled to the universal testing machine (EZ-SX/Shimadzu, Barueri, SP, Brazil) was used for the flexural strength test of 280 Mis (Fig. 3b). This device allowed the setting of each MI, which was gripped 3 mm before the transmucosal collar. The active chisel tip of the universal testing machine was positioned on one of the flat faces of the hexagonal head of the MI (Fig. 3b). Subsequently, a vertical force perpendicular to the MI long axis was applied to deform it by 1 mm (140 MIs) and 2 mm (140 MIs) (Fig. 3b). The active chisel tip of the universal testing machine operated at a speed of 0.5 mm/min [24].

In sequence, 70 MIs underwent morphological evaluation of their threads by scanning electron microscopy performed before insertion in artificial bone blocks and after removal. The artificial bone made of polyurethane (Sawbones Division of Pacific Research Laboratories, Vashon Island, Wash) was selected because it met the requirements of the American Society for Testing and Materials (F-1839-08), has been successfully used for biomechanical tests, and it is sold in different densities to better simulate the biomechanical characteristics of the bone tissues. A high artificial bone density (40 pcf) was used to evaluate whether different degrees of morphological damage occurs in SS-MI and TA-MI threads because of insertion procedure without previous bone drilling.

**Fig. 4:**
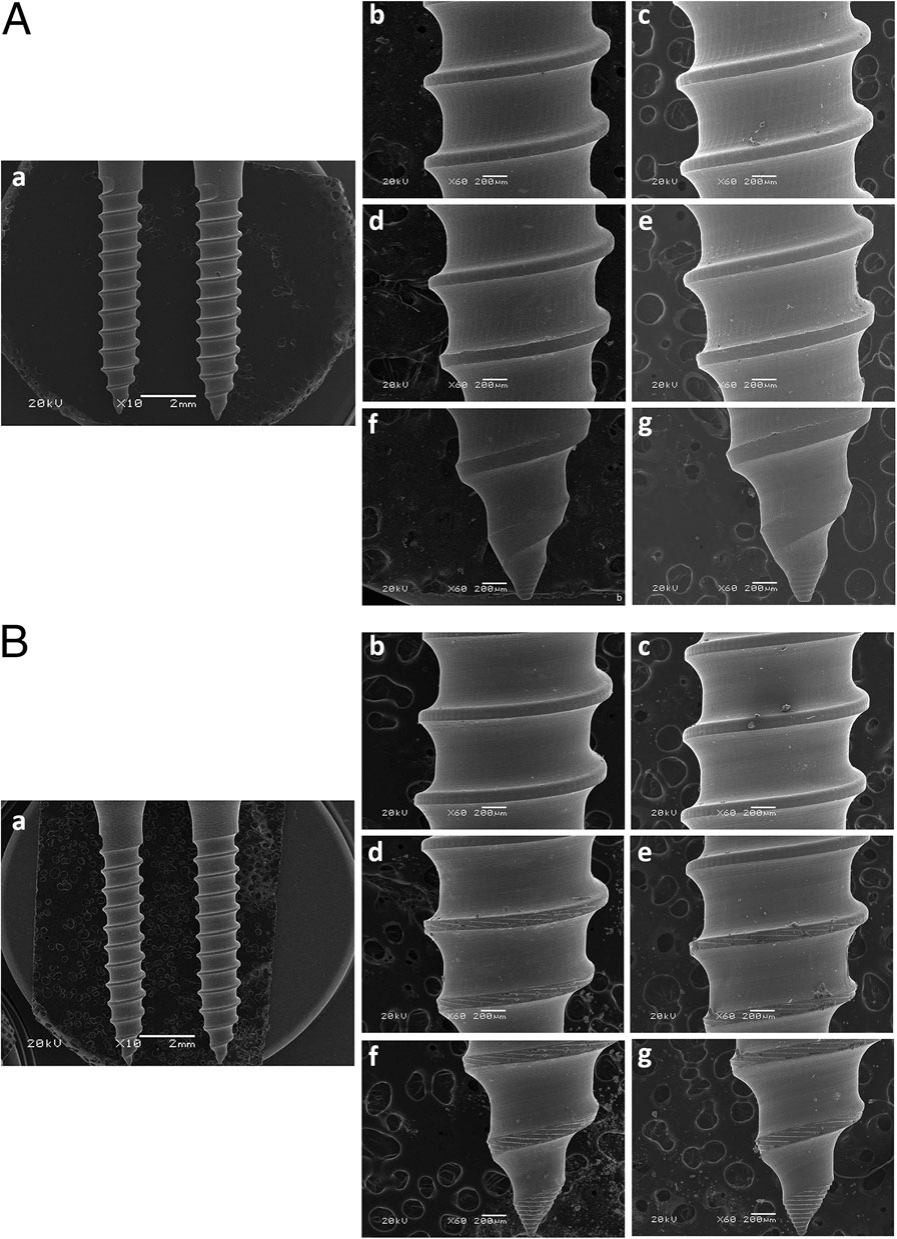
SEM analysis of the red side of 1.5 mm diameter SS-MI (**a**) and TA-MI (**b**). **a** × 10 magnification image showing the entire MI thread. **b**, **d**, **f** Pre-insertion evaluation at × 60 magnification of MI threads at H1, H2, and H3. **c**, **e**, **g** Post-insertion evaluation at × 60 magnification of MI threads at H1, H2, and H3

Prior to insertion into artificial bone, 70 as-received MIs were evaluated by a scanning electron microscope (SEM) operating at 20 kV (JSM6060/JEOL, Akishima, Japan). Before the initial microscopic analysis, each MI was stored separately in plastic containers identified by diameter and material. The hexagonal head of each MI had three of its six sides alternately marked with an overhead projector pen. Each marked side received a specific color: red side (RS), blue side (BS), and green side (GS). Subsequently, the MIs were mounted on aluminum sample holders for use in the SEM with a carbon double-sided sticky tape. The threads of the marked sides (RS, BS and GS) were evaluated at × 60 magnification and at three heights: height 1 (H1), involving the MI tip; height 2 (H2), involving two thread pitches above H1 and height 3 (H3), involving two threads pitches above H2. Thereafter, these MIs were manually inserted in and removed from the high-density artificial bone with the aid of a custom-fabricated device, which had a clamp to attach the artificial bone blocks and a parallelometer to drive the MI insertion and removal, preventing oblique placement forces. Finally, all MIs underwent second evaluation by scanning electron microscopy, which was guided by previous color marks performed on the MI head. Thus, the same MI threads evaluated at pre- and post-insertion stages could be visually compared and a qualitative analysis was performed (Fig. 4).

### Statistical analysis

A descriptive analysis of the data was performed obtaining means and standard deviations for the torsion and deflection tests. The Shapiro-Wilk test was used to verify the normality of the data.

Comparisons between the same diameter of SS-MIs and TA-MIs were performed using *t* tests for parametric and Mann-Whitney *U* tests for nonparametric data. The influence of each variable on the ranging of fracture torque and flexural strength was evaluated using multiple linear regression analysis.

Statistical analyses were performed with Statistica for Windows software (version 7.0; StatSoft, Tulsa, OK). The results were considered statistically significant at *P* < 0.05.

## Results

SS-MIs had higher values of fracture torque, with five of seven diameters presenting significant differences, while two diameters showed marginal non-significance (Table 1). The mean difference between the SS-MIs and TA-MIs fracture torque in the total sample was 4.09 Ncm, totalizing a 13.2% gain in torsional strength.

**Table 1:** Comparison of fracture torque between SS-MIs (*n* = 77) and TA-MIs (*n* = 77)

Diameters(mm)	Stainless steel (*n* = 11)		Titanium alloy (*n* = 11)		*p*
	Mean (N.cm)	SD	Mean (N.cm)	SD	
1.2	13.81	1.27	12.64	1.43	0.055^§^
1.3	15.15	2.92	12.90	1.60	0.008*^†^
1.4	25.94	1.66	18.40	3.07	< 0.001*^†^
1.5	27.19	1.78	24.79	1.04	< 0.001*^§^
1.6	35.32	1.84	31.81	1.77	< 0.001*^§^
1.7	44.37	4.24	40.83	4.13	0.061§
1.8	54.58	2.71	46.37	5.37	< 0.001*^§^

*Statistically significant at *p* < 0.05

†Mann-Whitney *U* test

§ *t* test for independent samples

The fracture torque was significantly influenced by diameter and metallic alloy (Table 2). The regression model explained 92.4% of the total fracture torque variation. The MI diameter explained 90.3% of the total variation in fracture torque, while the metallic alloy explained 2.2% (Table 2).

**Table 2:** Influence of MI diameter and metallic alloy on the fracture torque (multiple linear regression analysis)

Regression model ***n*** = 154	Unstandardized coefficients		Standardized Coefficients	***t***	***p***	Partial ***R***^**2**^	***R***^**2**^
	***B***	SE	Beta				
(Constant)	347.32	61.01		5.69	< 0.001*		0.924
Diameter	6.43	0.15	0.95	42.88	< 0.001*	0.903	
Metallic alloy	− 4.08	0.61	− 0.15	− 6.80	< 0.001*	0.022	

*Statistically significant at *p* < 0.05

SS-MIs had the flexural strength significantly higher than TA-MIs (Table 3). The mean difference between the flexural strength of SS and TA-MIs at 1 mm and 2mm-deflection in the total sample was 18.21 N and 17.55 N, respectively, totalizing a 20.2% gain in flexural strength.

**Table 3:** Comparison of flexural force between SS-MIs (*n* = 140) and TA-MIs (*n* = 140)

Diameter (mm)	Deformation (mm)	Stainless Steel (*n* = 10)		Titanium Alloy (*n* = 10)		*p*
		Mean(*N*)	SD	Mean(*N*)	SD	
1.2	1	31.27	3.21	27.94	1.95	0.012*^†^
1.3	1	43.68	3.06	38.34	2.39	< 0.001*^§^
1.4	1	64.56	2.82	43.93	2.40	< 0.001*^§^
1.5	1	83.10	4.57	60.12	1.42	< 0.001*^§^
1.6	1	98.83	5.24	68.13	5.75	< 0.001*^§^
1.7	1	119.95	9.93	95.64	10.22	< 0.001*^†^
1.8	1	129.89	17.14	109.63	12.17	0.007*^§^
1.2	2	42.70	5.70	36.73	2.00	0.005*^§^
1.3	2	51.78	2.40	47.60	3.85	0.006*^§^
1.4	2	78.32	2.57	54.54	2.93	< 0.001*^§^
1.5	2	95.50	2.88	72.63	2.70	< 0.001*^§^
1.6	2	101.86	5.04	83.06	4.22	< 0.001*^§^
1.7	2	137.79	8.58	112.87	4.87	< 0.001*^†^
1.8	2	162.17	9.67	139.84	7.41	< 0.001*^§^

*Statistically significant at *p* < 0.05

† Mann-Whitney *U* test

§ *t* test for independent samples

Flexural strength was significantly influenced by diameter, amount of deformation and metallic alloy (Table 4). The regression model explained 93.2% of the total flexural strength variation. The diameter explained 83.5% of the total variation in flexural strength, while the amount of deformation and metallic alloy explained 5.8% and 3.8%, respectively (Table 4).

**Table 4:** Influence of MI diameter, deformation, and metallic alloy on the flexural strength (multiple linear regression analysis)

Regression model ***n*** = 280	Unstandardized coefficients		Standardized coefficients	***t***	***p***	Partial ***R***^**2**^	***R***^**2**^
	***B***	SE	Beta				
(Constant)	1620.53	116.82		13.87	< 0.001*		0.932
Diameter	168.64	2.87	0.91	58.65	< 0.001*	0.835	
Deformation	14.45	1.15	0.19	− 15.55	< 0.001*	0.058	
Metallic alloy	− 17.88	1.15	− 0.24	12.56	< 0.001*	0.038	

*Statistically significant at *p* < 0.05

A total of 1260 scanning electron microscopy images were obtained from 35 SS-MIs and 35 TA-MIs, and qualitatively analyzed. In the visual analysis, it was not possible to observe any morphological damage to the MI threads or damage to the MI tip after insertion and removal in high-density artificial bone for either material. Figure 4a, b shows the similarity between the initial and final × 60 magnification images of two MIs with 1.5 mm diameter made of SS and TA, respectively.

## Discussion

To our knowledge, this is the first study comparing the mechanical strength of several diameters of SS-MIs and TA-MIs using a MI sample with standardized morphological features made by the same manufacturer, which could contribute with more reliable results. Indeed, when comparing SS-MIs and TA-MIs from different brands or morphological characteristics, several confounding factors may mask the influence of metallic alloys on the MI mechanical characteristics [2, 14].

In this study, the null hypothesis was rejected, because SS-MIs had higher mechanical strength. SS-MIs obtained a higher fracture torque than TA-MIs (Table 1). Five of the tested diameters showed statistically significant differences, and two diameters presented a borderline statistical similarity in relation to the adopted significance level (Table 1). Although there is no study comparing the fracture torque of SS and TA-MIs with a standardized sample, a previous study did not find significant fracture torque differences between different brands of MIs when the same diameter of SS-MIs and TA-MIs was compared [12]. These conflicting results may be due to the morphological and dimensional characteristics of thread (pitch, flank angle, thread form, thread depth, and taper), transmucosal and head of the MIs, which vary between different brands and may significantly influence the insertion and fracture torque [1, 2, 14, 25]. The influence of different brands on the fracture torque can be more readily observed in a previous study that evaluated 41 types of MIs from 11 different brands with diameters ranging from 1.3 to 2 mm. The results showed that MIs with the same diameter and different brands had a high variation (up to 18 Ncm) in the fracture torque [2].

SS-MIs had 13.2% more torsional resistance than TA-MIs. Although stainless steel produced an increase in torsional fracture, this beneficial effect should be carefully evaluated because it has a limited clinical impact. In fact, in the best scenario, the gain in mechanical strength provided by stainless steel was limited to an equivalent torsional resistance between a SS-MI and a 0.1 mm thicker TA-MI (Table 1). In fact, the multiple linear regression analysis showed that although the metallic alloy significantly influenced the torsional resistance of the MIs, only 2.2% of the fracture torque variation could be explained by this factor (Table 2). The MI diameter was the most influential factor (90.3%; Table 2). However, the increase in MI diameter is frequently limited by the anatomical characteristic and bone availability in the insertion sites. Thus, stainless steel MIs arise as an alternative to clinicians to reduce fracture risk, especially when bone availability does allow a significant increase of MI diameter until reaching a safer thickness (1.5 mm) and/or the bone quality requires a MI with greater torsional strength (mandibular buccal shelf) [1, 16].

The mean force applied to achieve 1 mm and 2 mm-deflection was significantly higher for SS-MIs than for TA-MIs (Table 3). A previous study demonstrated that low threaded SS bone screws had 46.5% more bending resistance than the low threaded TA bone screws, and the stiffness of SS bone screws was higher than that of TA bone screws [26]. However, our findings were more modest, showing that on average SS-MIs had 20.2% more bending resistance than TA-MIs. It has been suggested that commercially available SS-MIs withstand greater torsional and flexural force magnitude compared to other commercially available TA-MIs mini-implant systems [11]. However, comparison of different MI geometric designs and brands to evaluate the role of MI composition in its mechanical strength can be a wasted effort due to the influence of several confounding factors in the results [2, 12, 14, 26]. Although our findings confirm that SS-MIs can withstand greater torsional and flexural force magnitude, they should be considered with caution when extrapolated to commercially available brands. After all, the SS-MI gain in mechanical strength is not so impactful to avoid that SS-MIs and TA-MIs with similar diameter but different brands and designs have similar mechanical strength as observed in previous study [12].

Similar to torsion test, in the best scenario, the gain in mechanical resistance provided by stainless steel was limited to an equivalent flexural strength between a SS-MI and a 0.1-mm thicker TA-MI (Table 3). The multiple linear regression analysis showed that the flexural force required for 1 mm and 2-mm-deflection was significantly influenced by diameter, amount of deformation and type of metallic alloy (Table 4). The regression model explained 93.2% of the total variation in the force applied to deflect MIs (Table 4). The diameter of the MIs explained 83.5%, while the amount of deformation and the type of metallic alloy explained 5.8% and 3.8%, respectively. The results reflect the limited gain in flexural strength associated with MIs made of SS. However, this structural reinforcement provided by SS may have an beneficial impact in surgical techniques involving an intentional flexural force due to changes in mini-implant insertion path as during placement of infrazygomatic bone crest screws [16]. In addition, currently, mini-implants have been frequently used to anchor orthopedic forces for skeletal malocclusion correction, which could be better supported by the extra mechanical strength provided by SS-MIs [27–29].

The MI area selected for qualitative evaluation by SEM was based on the greater structural weakness of the MI tip and the high mechanical stress applied to this area during self-drilling insertion [30]. In the visual analysis of SEM images, the retrieved MIs of both material (SS and TA) did not show any noticeable deformation, fracture or other morphological damage in their threads and tips (Fig. 4a, b). However, it has been demonstrated that corrosion and interactions with body fluids and tissues may significantly influence the morphological changes and damages observed on the surface of retrieved MIs [31]. Considering that SS-MIs are more prone to corrosion [32], studies should be performed to evaluate its impact on morphological surface changes in SS-MI retrieved from patients. A limitation of this study was the lack of chemical composition analysis of the as received MIs, which could be used as a parameter for qualitative analyses with future studies and commercial brands.

### Clinical implications

Although SS-MIs can have lower manufacturing cost and higher mechanical strength, they should not be seen as a substitute for TA-MIs [22], but rather, as a clinical alternative to TA-MIs, since SS-MIs seem to have similar or lower success rates than TA-MIs [17–19, 23, 33, 34]. A recent systematic review suggested similar clinical efficiency of SS-MIS and TA-MIs [15]. However, two of six studies qualified for this review showed that TA-MIs had a higher success rate [23, 33]. Whether these results are directly associated or not with the greater biocompatibility of the titanium alloy remains to be clarified. A preliminary study reported that there were no differences in histologic responses between SS-MIs and TA-MIs [20]. While stronger scientific evidences comparing the success rate of SS-MIs and TA-MIs are not available, SS-MIs should be indicated for insertion sites that require a greater torsional and flexural strength (mandibular buccal shelf and infrazygomatic crest) and insertion sites with limited bone availability requiring mini-implant diameter thinner than 1.4 mm, especially if drill-free insertion is planned [1, 16, 35]. In addition, the use of SS-MIs could be considered when bone screws are used to anchor high orthopedic forces as in miniscrew-assisted rapid palatal expansion (MARPE) due to an increased flexural load [36].

## Conclusions

The null hypothesis was rejected because of the following:

- In general, SS-MIs had a higher torsional strength than TA-MIs.
- SS-MIs had higher flexural strength than TA-MIs.
- On average, SS-MI had 13.2% and 20.2% more torsional and flexural strength than TA-MIs.
- The use of SS-MI can reduce the fracture risk without increasing the MI diameter.
- In the total sample, variation in torsional and flexural strength were more influenced by the diameter (90.3 and 83.5%) than by the type of metallic alloy (2.2 and 3.8%).
- The threads of SS-MIs and TA-MIs had similar resistance to morphological damages after insertion in high-density artificial bone.

## Data Availability

The datasets used and/or analyzed during the current study are available from the corresponding author on reasonable request.

## Abbreviations

MI: Mini-implant
SS: Stainless steel
TA: Titanium alloy
SS-MI: Stainless steel mini-implant
TA-MI: Titanium alloy mini-implant
SEM: Scanning electron microscopy
Ti-6Al-4V: Titanium grade 5 (6%Al–4%V)
PCF: Pounds per cubic feet
RS: Red side
BS: Blue side
GS: Green side
H1: Height 1
H2: Height 2
H3: Height 3
